# Strongly coupled plasmon-exciton polaritons for photobleaching suppression

**DOI:** 10.1515/nanoph-2024-0259

**Published:** 2024-08-28

**Authors:** Justina Anulytė, Vytautas Žičkus, Ernesta Bužavaitė-Vertelienė, Daniele Faccio, Zigmas Balevičius

**Affiliations:** 226274 Department of Laser Technologies, Center for Physical Sciences and Technology, Vilnius 10257, Lithuania; School of Physics and Astronomy, University of Glasgow, Glasgow G12 8QQ, UK

**Keywords:** plasmon exciton coupling, strong coupling, photobleaching, polaritons

## Abstract

Strong light–matter interactions have received a lot of attention, for example in the pursuit of plasmonic-excitonic structures as coherent light sources with low-power threshold. In this study, we investigate the influence of room temperature strong coupling between surface plasmon polaritons (SPP) and excitons on fluorescence lifetimes and photobleaching effects. Our plasmonic-photonic structure, comprising of thin silver (Ag) and gold (Au) layers with a Rhodamine 6G (R6G) dye layer, shows a clear shift in the plasmon resonance and R6G absorption lines with varying incident angles, indicative of strong coupling, with a measured Rabi splitting of approximately 90 meV. Fluorescence lifetime imaging microscopy (FLIM) was then employed to assess photobleaching, revealing a significant reduction in photobleaching effect for in strongly coupled plasmonic-excitonic structures compared to single Rhodamine R6G layers. Our findings indicate the pivotal role of strong light–matter interactions in reducing photobleaching effects and stabilizing fluorescence intensities, offering promising avenues for developing quantum multiparticle nanophotonic devices with enhanced stability and performance.

## 1 Introduction

Strong light matter interaction in photonic structures with coupled excitons and plasmons has emerged in coherent light source research due to the small power threshold required to achieve plasmonic lasing [1]–[3]. The strong light–matter interaction of molecules placed in the cavity can modify their emission [4]–[7]. The strong coupling regime then manifests as the splitting of energy spectra at the anti-crossing point. Furthermore, photonic structures, based on strong plasmon-exciton coupling could allow improving the operation speed of nanoelectronics in integrated circuits at room temperature [8], [9]. The consequence of strong light–matter interaction in the hybrid states of organic molecules and cavity polaritons is the extended coherence [10] properties. This is in contrast with weak coupling, where only the spontaneous emission can be modified. This behaviour is also linked to the observation that strong coupling occurs when light–matter interaction rate is faster than the energy losses in the system, therefore, the energy exchange between emitter and plasmonic cavity occur during a coherence time [11], [12]. Recently, there has been an increase in the use of plasmonic nanocavities to achieve strong coupling due to their ability to highly localise electromagnetic fields at the metal-dielectric boundary and the potential to surpass the diffraction limit due to the small cavity volume [13], [14]. This leads to a small cavity volume which is desirable despite the fact that metals have high Ohmic losses and the quality factor (Q-factor) is rather low compared with purely photonic cavities [15], [16]. The important feature of strong coupling between cavity and organic emitter is that this interaction regime significantly changes the chemical reaction rates of molecules participating in the strong coupling at room temperature [4], [17]. One of the fundamental phenomena appearing in many organic molecules is the photobleaching effect, which can suppress the fluorescence of the dye molecule due to the transition of singlet excited state electrons to the triplet state. Furthermore, the fluorescent molecule can suffer from irreversible photo damage, which reduces the number of emitters over time. It was shown that strong coupling between localized plasmons of gold nanoparticles and organic dye molecules can significantly suppress photobleaching [18], [19]. One of the first theoretical investigations on how strong coupling influences photobleaching in organic molecules were performed by Galego et al. [19]. They found that in the strong coupling regime, many-particle systems suppress photobleaching more efficiently than single strongly coupled organic molecule systems. Later it was experimentally shown by Munkbhat et al. [18] that localized surface plasmon polaritons in Ag nanoparticles with J-aggregates under different coupling strengths influence the photobleaching, they have found that a larger Rabi gap suppresses photobleaching more efficiently. In contrast to these two papers, we perform experimental studies of photobleaching suppression and fluorescence lifetime measurements on planar nanostructures consisting of thin organic dye molecule layer and metallic film. Optical excitation can create singlet excitons, but subsequent transition from the singlet to the triplet dark state can occur due to weak spin–orbit coupling and undergo the intersystem crossing (ISC). The lifetime of the triplet state is longer than that of the singlet, therefore the probability to interact with environmental oxygen is high. The interaction of environmental oxygen with triplet exciton occurs via charge transfer mechanism and as a result creates reactive oxygen species which chemically destroy the fluorophores. Photobleaching in turn influences many physical–chemical processes and can have a negative impact on the investigation of various systems in which photoluminescence effect may occur [20]. The decrease of fluorescence emission intensity due to photobleaching is commonly reported and thin metal layers or metallic nanostructures have been widely used to enhance the emission [21] due to plasmonic effect that induces strongly localized electric field. The luminescence enhancement can be achieved by two mechanisms – the light excitation and emission. The incident light is absorbed by the molecule (e. g. fluorescent organic molecule), however, when a metal nanoparticle is in the vicinity of the molecule, the light is coupled to the confined local surface plasmon (LSP) field and the energy is then absorbed by the molecule. Indeed, optical energy is coupled to the tightly confined plasmonic field in the vicinity of organic molecule enhancing the absorption rate of the molecule. The process depends on the absorption cross section, and the relaxation of the excited molecule radiative and nonradiative decay pathways, where the radiative decay outcome is the relaxation by emission at plasmon frequency (*ω*_sp_). The photoluminescence is enhanced when the emission and excitation frequencies are close to the plasmon resonance and the nonradiative decay rate is smaller than the radiative decay. Furthermore, such absorbed energy can be emitted with enhanced spontaneous emission of excited molecules through the Purcell effect [22] (weak coupling regime). The rate of photochemical reactions can be modified by the weak coupling in plasmonic systems. However, the relaxation pathways are considerably smaller in weak coupling compared to the strong coupling regime. However, we underline that weak coupling with plasmonic modes does not inhibit the photobleaching effect.

In this work we investigate the influence of strong coupling on photobleaching and fluorescence lifetimes of rhodamine 6G dye in nanostructures that support plasmon-exciton polaritons states. The plasmonic-photonic nanostructures used in this research consisted of thin silver-gold films (Ag-Au) with rhodamine 6G dye in nanometre thickness layers. Total internal reflection ellipsometry (TIRE) was used for the plasmon-exciton polariton system by selectively filtering out the incident light responsible for exciton component generation in the hybrid polaritonic mode and thus prove the strong coupling. We then use fluorescence lifetime imaging microscopy (FLIM) to demonstrate that the strong coupling between surface plasmon polariton (SPP) and dye excitons plays a pivotal role in suppressing photobleaching.

## 2 Methods

### 2.1 Sample fabrication

The sample used for the hybrid surface plasmon-exciton polariton mode excitation consisted of two thin metal layers: silver (Ag, ∼35 nm) and gold (Au, ∼9 nm) with a top thin layer dye rhodamine 6G (R6G) and polymer polymethyl methacrylate (PMMA) layer (PMMA-R6G, ∼20 nm). The Ag and Au films were deposited on a BK-7 glass substrate using magnetron sputtering method (Kurt J. Lesker PVD 225), the PMMA-R6G was deposited on the top of the metal layers using the spin-coating method (at 3,000 rpm from solution). For obtaining solid state matrix of rhodamine 6G dye PMMA was used, both of which were dissolved in ethanol. In the preparation of our samples, PMMA and the dye (R6G) were mixed in ethyl alcohol at a ratio of 3:1. This ratio refers to the volume of the solutions of PMMA and R6G in ethyl alcohol. Specifically, 3 parts of PMMA solution were mixed with 1 part of R6G solution. The final concentrations mentioned in the paragraph represent the concentrations of the solutions prior to mixing the two solutions at a 3:1 ratio before the spin-coating process. To be precise: the PMMA solution concentration was 1 × 10^−5^ mol/L (*c*_m_ = 0.0367 g/L) and R6G solution concentration was 25 × 10^−3^ mol/L. The mixture of these solutions was then spin-coated onto the metal surface to form the thin film, where the film, once dried, had a thickness of approximately 20 nm. A reference sample consisting of glass cover slip with PMMA-R6G layer spin-coated with the same parameters (3:1 ratio) as in Ag-Au sample was produced. To distinguish the photobleaching properties in weak and strong coupling for the structure with thin metal layer the third sample was produced by inserting spacer layer of 20 nm PMMA between Ag-Au metal layer and PMMA-R6G layer (Ag-Au(44 nm)/PMMA(20 nm)/PMMA-R6G(20 nm)). The last two described samples were used as a reference to evaluate the photobleaching of pure PMMA-R6G and Ag-Au/PMMA-R6G with spacer in weak coupling regime.

### 2.2 Total internal reflection ellipsometry

**Figure 1:**
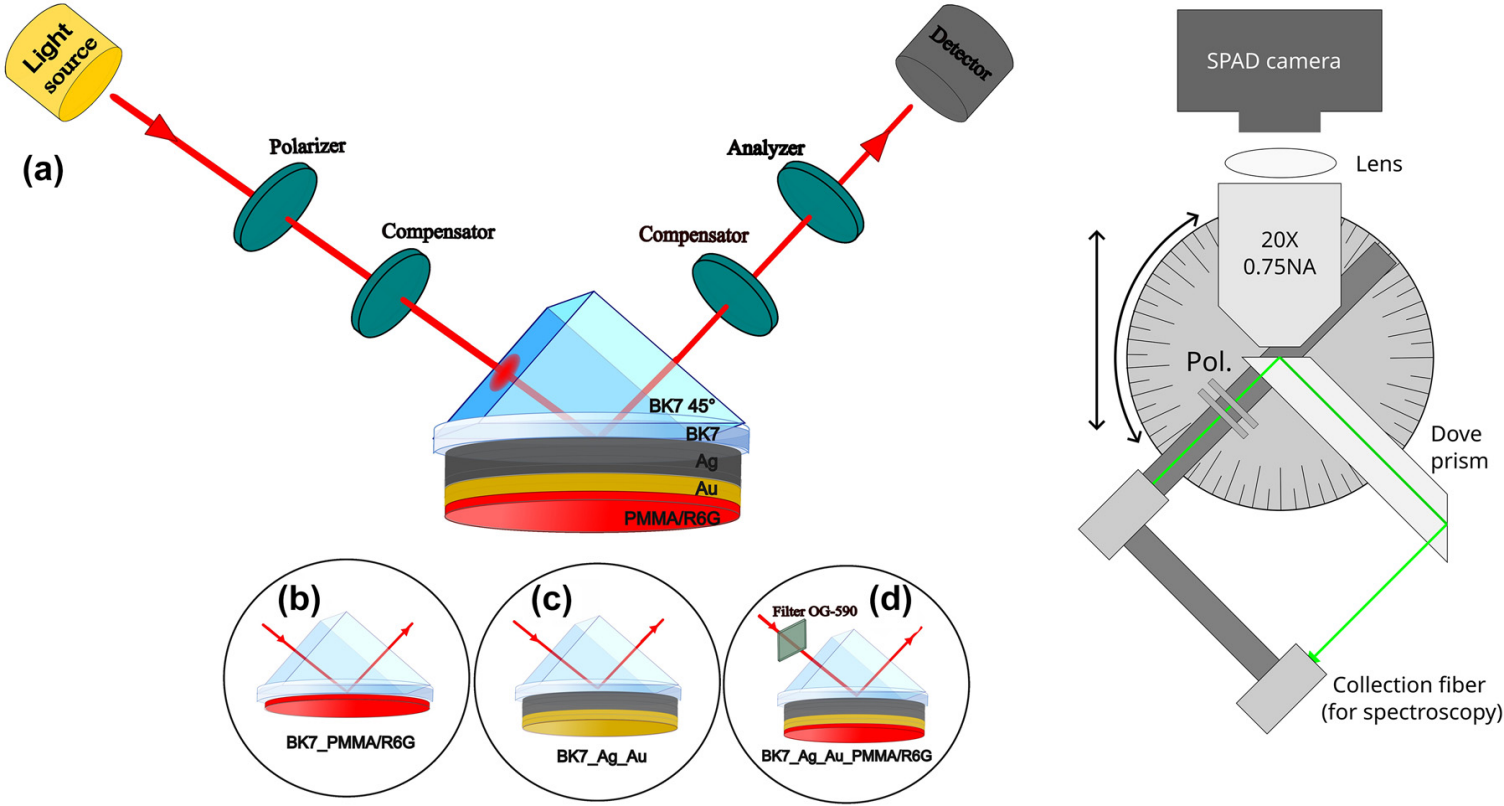
TIRE scheme (left) of excitation configurations for the (a) hybrid exciton-SPP mode, (b) R6G absorption lines, (c) single SPP mode and (d) the hybrid exciton-SPP mode with an optical filter OG-590. Schematic of the dove-prism TIRF system (right) with illumination/collection arm that is attached to a rotation and translation stage. This arm is independent of the dove prism, and the microscope objective.

The R6G embedded in PMMA layer and metal/dye structure described above were measured using spectroscopic ellipsometry (SE) and total internal reflection ellipsometry (TIRE). The spectroscopic ellipsometer used for these measurements was a J. A. Woollam RC-2 with two rotating compensators. The light source was a combined deuterium/quartz-tungsten halogen lamps with a spectral range of 210–1700 nm. For the excitation of the hybrid surface plasmon-exciton polariton mode, a total internal reflection (TIR) configuration of spectroscopic ellipsometry with a 45° prism (made of BK7 glass) coupler was used (Figure 1).

Four different ellipsometric spectra were measured. Firstly, the ellipsometric spectra of Rhodamine embedded in PMMA matrix measurements were performed from which the absorption lines (Figure 1(b)) of R6G were determined. The second measurement was performed in TIRE configuration with Ag-Au layer on the glass plate attached through the immersion oil (BK7 matched) to the BK7 glass prism for investigation of optical dispersion of excited SPP (Figure 1(c)). Next, TIRE spectra of Ag-Au/PMMA-R6G were measured (Figure 1(a)), where both components (exciton and SPP) manifested themselves at the 470–730 nm range of wavelengths, respectively. In order to prove that the anti-crossing of dispersion curves of SPP and R6G dye exciton is caused by strong coupling, an optical filter (Figure 1(d)) was used to excite only one of the resonance components. To achieve this an optical filter Schott OG-590 long pass was used to cut a part of the white light in order to leave only the SPP resonance.

### 2.3 Dove prism total internal reflection fluorescence microscopy and spectrometry

An in-house built system was used for FLIM and spectral reflection intensity measurements (Figure 1 (right)). The system consists of a BK7 dove prism, a microscope focus body (not shown in Figure 1), and an illumination/detection arm. The illumination arm can rotate/translate independently of the microscope objective (NIKON 20× 0.75NA) and the dove prism. This means that for small angles used in this work, only one collection arm is necessary when spectral measurements are required (in contrast to two arms in ellipsometry systems for example). Spectral measurements were performed using OceanOptics USB4000-UV-VIS spectrometer, with Thorlabs SLS201L/M stabilized tungsten-halogen light source. FLIM measurements were carried out using SuperK EXR-20 ps pulsed supercontinuum source filtered with 520–10 nm filter (Thorlabs FBH520-10) and coupled into a single-mode fiber. The fluorescence (filtered with a 550 long-pass filter) was imaged using Horiba FLIMera single photon avalanche diode array. The sample was coupled to the prism using immersion oil (*n* ≈ 1.51). Exposure was set to 30 s, and the sample was continuously illuminated between acquisitions (spaced 1 min apart). The average power density used was ∼40 mW/cm^2^. The instrument response function (IRF) of the system was measured using light reflected from a bare coverslip, with the long-pass filter removed. Because each single photon avalanche diode (SPAD) in the array has a slight temporal offset, the recovered data were temporally aligned in post-processing to allow averaging the data across all pixels.

## 3 Results and discussion

**Figure 2:**
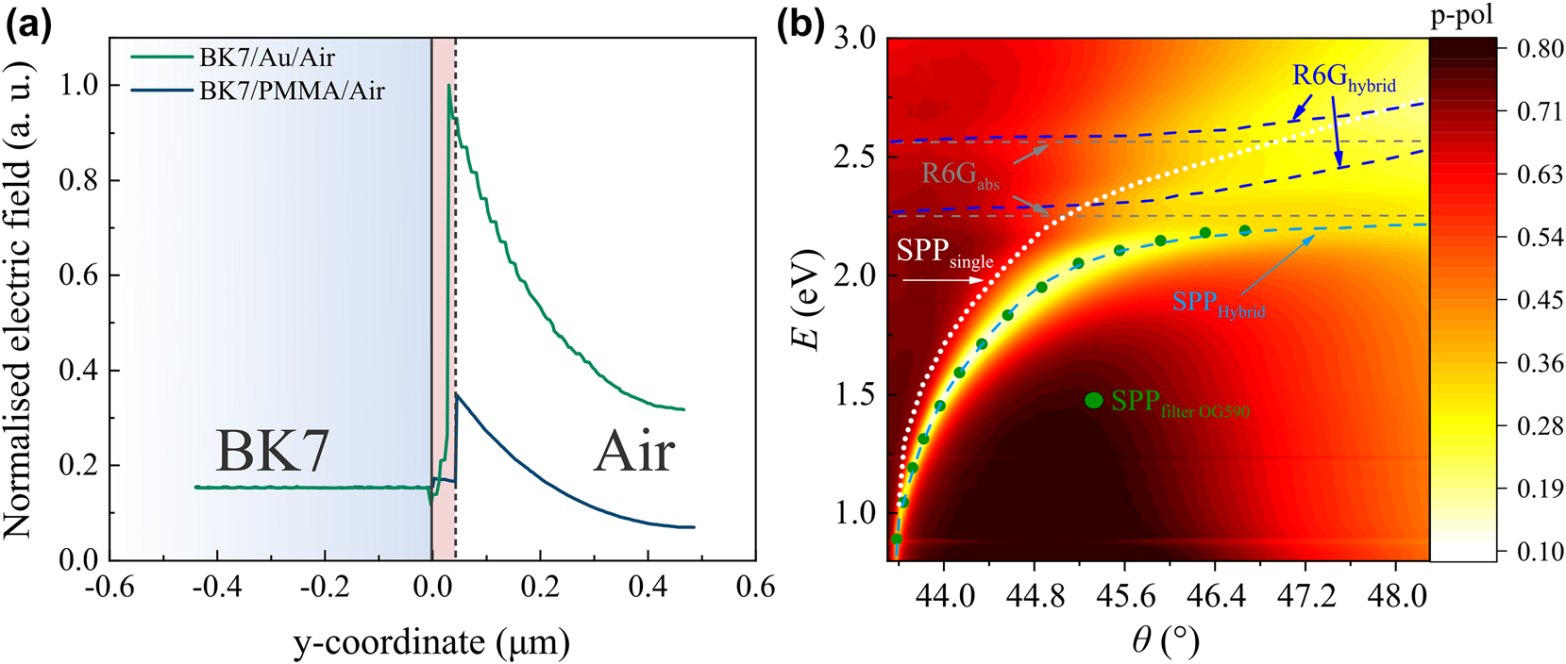
The modelled normalised electric field distribution of metal/air (green) and PMMA/air (blue) structures (a). The dispersive relationship of hybrid mode (dark blue (R6G) and light blue (SPP) dashed curves) and single SPP (white dotted curve) and excitons R6G (grey dashed lines) by total internal reflection ellipsometry method (b). The measurements with filter OG-590 are marked as green dots.

To achieve the strong coupling regime the interaction between emitter and optical environment should be strong enough to modify the energy levels responsible for emission. SPPs are able to confine energy into small volumes at the metal-dielectric interface, thus, the enhanced electric field can interact with emitter strongly enough. The near field character of SPP is related with the fact that such modes are bound to the interface. The confinement of the optical field in the vicinity of the interface arises from the evanescent nature of SPP. The near-field character determines the propagation and localization properties of the SPP’s, as well as, increased possibilities for strong coupling between emitters and SPP’s. In order to compare the electric field strength of SPP (metal/air) and evanescent wave at dielectric/air interface two structures were modelled, where either Au (45 nm) or PMMA (45 nm) were embedded between two semi-infinite materials – BK7 glass and air. Both of the samples were investigated under total internal reflection (TIR), where AOI was equal to 45°. As can be seen from Figure 2(a), there is a peak field enhancement in the metal film (green line) case that is approximately 2.9 times stronger compared to the bare film sample (blue line). In case of a thin metal film the excitation of SPP works as an open cavity in which organic dye emitter is placed.

The TIRE method was used for analysis of the dispersion and optical properties of single SPP for structure consisting of BK7 glass substrate and thin Ag-Au layer. The single SPP dispersion is shown in Figure 2(b) marked by white dotted curve. In order to determine the absorption lines of the dye, the PMMA-R6G layer was deposited on BK7 glass substrate and measured by spectroscopic ellipsometry. From the measured ellipsometric spectra the absorption lines of the PMMA-R6G were determined (*λ*_1_ = 482 nm, *λ*_2_ = 551 nm) and are represented by the grey dashed in Figure 2(b).

In order to analyse the influence of the dye exciton on the optical response of the Ag-Au/PMMA-R6G structure the sample was attached to the BK7 glass prism via refractive index matching liquid and measured in TIRE configuration. First, the TIRE spectra of Ψ and Δ ellipsometric parameters for hybrid surface plasmon-exciton polariton structure were measured. The results are presented in Figure 2(b) as reflection intensity maps of the *p*-polarized light versus energy in the range of angle of incidence *θ* = [43–48°]. As can be seen from Figure 2(b) the hybrid exciton-SPP mode (marked by dark blue and light blue dashed lines) shifts and bends from the initial values of the single exciton (grey dashed lines) and single SPP (white dotted curve) modes.

In order to prove that the SPP resonance and dye excitons are strongly coupled, we used TIRE with spectral filtering where we filtered out one of the hybrid mode components. This method was previously used to prove strong coupling between the two components of a hybrid mode of strongly coupled plasmonic resonances [23]. Here we use this method to identify the strong coupling between plasmonic resonance and R6G dye excitons.

The ellipsometric parameters of the sample in TIRE configuration (Ag-Au/PMMA-R6G) were measured without an optical filter (Figure 2(b) dark blue and light blue dashed lines) and with an OG-590 filter (Figure 2(b) green dots) in order to determine if the exciton and SPP mode is in strong coupling. The spectra measured with optical filter OG-590 are indicated as green dots in Figure 2(b), where the filter cuts out a part of light incident onto sample leaving only the SPP component of the hybrid mode. The use of filters allows to distinguish if the system is in strong coupling or interference of two excitations. The interference can lead to a significant contribution in the optical response as a result leading to a distorted optical response, for example Fano resonance [24]. Since the experimentally measured dispersion of the SPP with the filter in place follows the unfiltered dispersion of SPP component, it indicates that the system is indeed in strong coupling.

It was shown that the strong coupling between J-aggregates of dye molecules and gold nanoparticles can suppress photobleaching [18]. A fluorophore with a high ISC quantum yield can transfer a significant amount of its population to the long-lasting triplet state. This is because the molecules interact with the environment in the triplet state, which in turn leads to photobleaching. Fluorescence lifetime imaging microscopy (FLIM) method is independent of the concentration of the fluorescent species. However, typical FLIM samples can have a number of different fluorophores that can be photobleached at different rates, thus, the shape of decay signal changes [25], [26]. Moreover, the effect of photobleaching in FLIM can be enhanced due to high optical energy in the light pulses which are necessary for the required large number of photons in order to ensure acceptable signal to noise ratio. Thus, FLIM can create reactive oxygen species that can result in an increased number of damaged fluorophores. In this research, FLIM was used for the investigation of influence of light–matter strong coupling to the suppression of photobleaching in R6G dye exciton. FLIM signal detects all types of emitting fluorophores, (various types of fluorophores with different lifetimes that interact differently with the environment) so the photo-bleaching rates are different and the fluorescence decay changes. However, when strong light–matter interaction takes place the singlet probability to interact with environmental oxygen is strongly suppressed, therefore this process influences the fluorescence lifetime decay.

**Figure 3:**
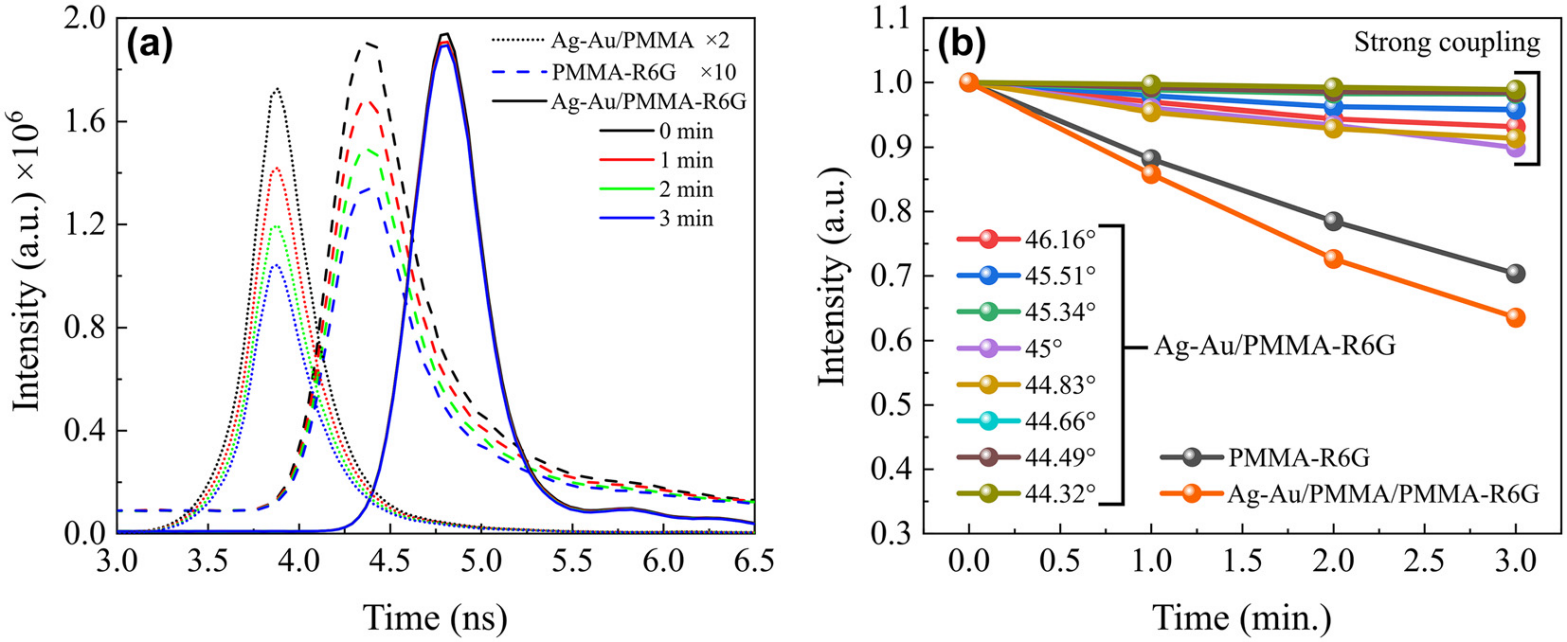
Fluorescence lifetime intensity dynamics in time. (a) Fluorescence intensity changes in time (from 0 to 3 min) due to photobleaching effect of R6G in plasmonic-nanophotonic structure (solid curves) structure with spacer between Ag-Au and PMMA-R6G (dotted lines) and of reference (dashed curves) samples. The AOI for plasmonic structure shown is 44°. FLIM curves of PMMA-R6G on the coverslip (AOI = 45°) are multiplied by a factor of 10 and structure with spacer by factor of 2 in order to show the counts at the same scale as the plasmonic-nanophotonic structures. (b) Photobleaching evolution in time of BK7/Ag-Au/PMMA-R6G structure at different incident angles, and a glass substrate with single R6G dye layer of (black) at 45° AOI.

In order to demonstrate the influence of photobleaching on the fluorescence lifetime two samples were used: (i) a fluorescent molecule layer and (ii) a plasmonic structure with fluorescent molecule layer. Firstly, a conventional sample of BK7 glass substrate with PMMA-R6G layer on top was measured (Figure 3(a) dashed lines). As the bare PMMA-R6G layer absorption lines are determined by the organic fluorescent molecules which absorb at 482 nm and 551 nm and they are non-dispersive at different angles of incidence, these samples were measured at the 45° AOI in the Kretchmann configuration (with glass prism coupled with immersion oil). It can be seen that the fluorescence lifetime intensity peak decreases with increasing laser exposure time. For the second sample Ag-Au/PMMA-R6G was illuminated in the θ = 44–46° AOI range to optically excite the strongly coupled states between SPP and R6G dye exciton (Figure 3(a) solid lines). As can be seen from Figure 3(a) (44°), the R6G intensity peak now experiences significantly less photobleaching in time compared to the sample with only the PMMA-R6G layer. In order to evaluate the photobleaching effect on the fluorescence lifetime peak intensity, the change of the photo-bleaching effect over time was analysed by altering the angle of light incidence (Figure 3(b)). The intensity of the curves of the Ag-Au/PMMA-R6G structure decrease much slower than intensity of PMMA-R6G sample (Figure 3(b) black circles). The fluorescence lifetime intensity of Ag-Au/PMMA-R6G sample drops only by approximately 5 % in 3 min, compared with 30 % of single PMMA-R6G layer.

In order to prove the influence of the strong coupling effect to the suppression of photobleaching, we performed measurements with the sample where a 20 nm PMMA spacer is embedded between metal and PMMA-R6G layers (Ag-Au/PMMA/PMMA-R6G structure in Figure 3(a) and (b)). The fluorescence lifetime intensity of sample with PMMA spacer in 3 min period decreases by similar amount as in single PMMA-R6G case. This clearly shows the influence of the strongly coupled plasmon and exciton on the reduction of photobleaching effect (6-fold reduction) at the zero detuning point and dependence on the AOI. These results indicate that strong coupling interactions play a crucial role in stabilizing photo-bleaching mechanisms and open possibilities for creating nanophotonic devices with organic molecules. Additionally, the improvement FLIM signal using nanostructures in the strong coupling regime provides the opportunity to investigate molecular relaxation processes without the influence of photo bleaching.

Note that the IRF of the system is limited to ∼150 ps. The data is fitted using least-squares approach, where the model function is a convolution between the IRF data, and a bi-exponential decay equation [27]:

$$I=a_{1}\exp\left(-\frac{t-t_{0}}{\tau_{1}}\right)+\left(1-a_{1}\right)\exp\left(-\frac{t-t_{0}}{\tau_{2}}\right),$$

where *I* is intensity *τ*_1_ and *τ*_2_ are the two distinct lifetimes, *a*_1_ is the decay amplitude of the first lifetime, *t* and *t*_0_ are the time and temporal offset, respectively.

While FLIM literature suggests that lifetimes that are ∼10× shorter than the IRF can be recovered, the recovered values must be interpreted with care. Nonetheless, there is a clear difference between the decay curves with strongly coupled plasmon-exciton polaritons and pure exciton on the sample without metal. Due to the use of TIR configuration, that is required to achieve SPP, the surface plasmon electric field is localized at the metal/PMMA-R6G interface. It is reasonable to assume that the main contribution to lifetimes comes from the molecules near the interface strongly coupled to the surface plasmon electric field. The enhancement of fluorescence lifetime intensity arises due to the suppression of the photobleaching effect, caused by the strong interaction between the plasmon and exciton, which influence the shortening of the fluorescence lifetime of R6G molecules. This is also confirmed by the lifetime’s fitting procedure for the sample with a metal layer (strongly coupled) where the obtained values of amplitude *a*1 were close to 1 along with the lifetime *τ*_1_ being significantly shorter than *τ*_2_ (Table 1). Meanwhile, for the sample without a metal layer the fitting parameter *a*_1_ was considerably lower.

**Table 1:** Components of lifetime decay from bi-exponential fits of data shown in Figure 4.

Fit	*a*_1_	*τ*_1_ (ns)	*τ*_2_ (ns)	*R* ^2^
Ag-Au/PMMA-R6G 44°	0.972 ± 0.0058	0.079 ± 0.0035	0.732 ± 0.1298	0.98
Ag-Au/PMMA-R6G 47°	0.995 ± 0.0008	0.037 ± 0.0009	1.701 ± 0.3209	0.994
CS/PMMA-R6G 45°	0.899 ± 0.0104	0.077 ± 0.0038	0.585 ± 0.0498	0.983

**Figure 4:**
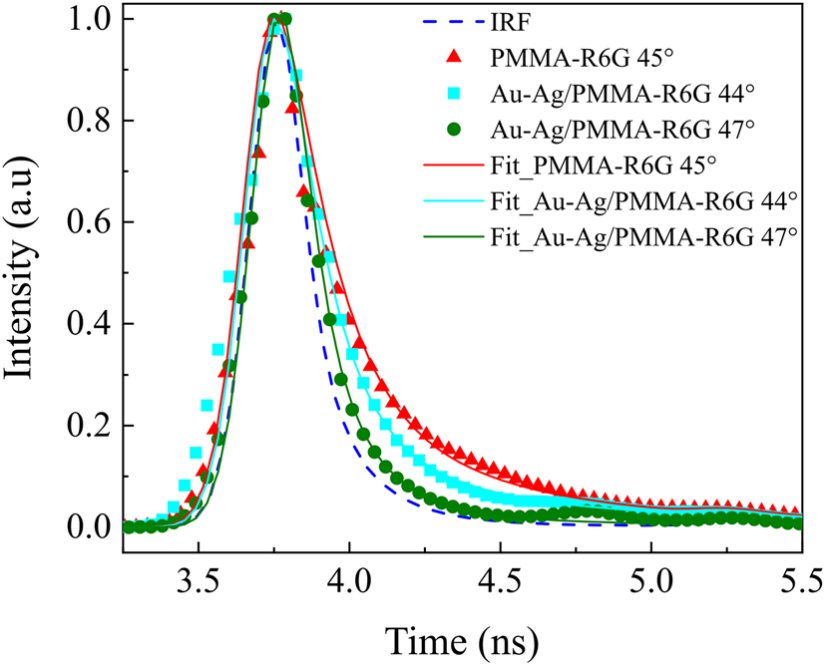
Fitted normalized fluorescence lifetime decay curves for different samples (solid lines), as well as the instrument response function (IRF). Nanostructures (Ag-Au) with a layer of spin-coated PMMA-R6G on have a significantly larger fraction of short lifetime component compared to the control sample (CS_PMMA-R6G 45°). In fact, nanostructure samples show a significant contribution from very short lifetimes (limited by the IRF of the system).

**Figure 5:**
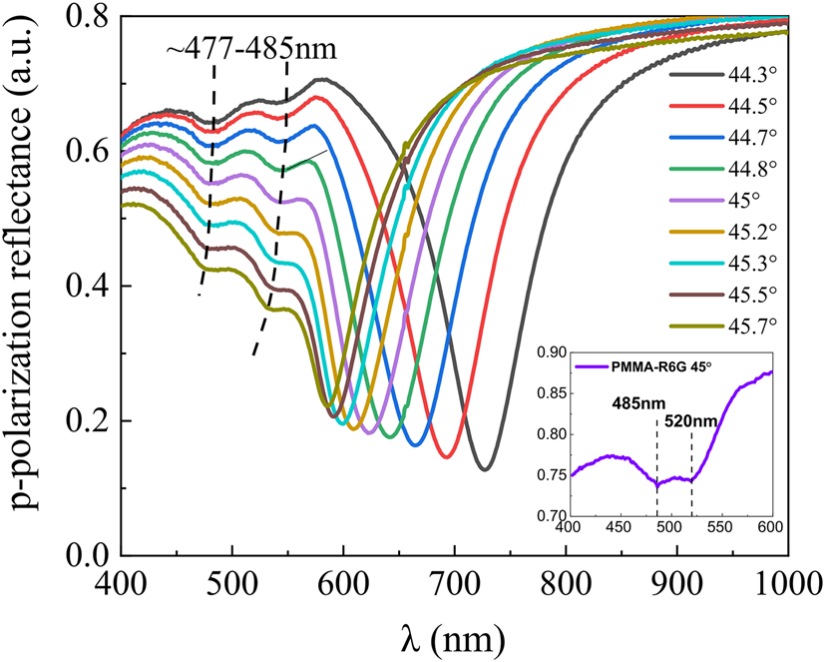
The experimental *p*-polarization reflectance (at 44–46° AOI) spectra of a hybrid SPP-exciton mode (black dashed lines show the R6G absorption lines variation with AOI). The inset on the bottom right shows the R6G absorption lines (at 45° AOI). The dashed lines correspond to the hybrid SPP-exciton mode component of PMMA-R6G absorption.

Each of the obtained fluorescence decay curves (Figure 4) correspond to a certain *p*-polarized reflectance spectra taken from ellipsometric measurements (Figure 5). While the fluorescence decay can be easily determined from fluorescence lifetime measurements, the influence of strong interaction between SPP and dye exciton components can be inspected in the frequency domain measurements. Spectroscopic ellipsometry measurements in TIRE seen in Figure 5 were performed at the angles of incidence between 44 and 46°. The corresponding hybrid SPP-exciton resonances appear, in the vicinity of the zero-detuning point, where strong interaction between SPP and R6G dye was achieved. From the graph (Figure 5), it is evident that as the incident angle increases, the lower polariton branch (LP) shifts towards the blue side of the spectra. Together with LP component the upper polariton branches (UP), also shift towards shorter wavelengths and bend (black dashed lines), contrary to R6G dye absorption lines (marked with dotted black lines in Figure 5 inset). The inset in Figure 5 shows the *p*-polarized reflectance spectra of a single dye layer (PMMA-R6G) on microscopic glass substrate. The spectral measurements have shown that the R6G dye absorption lines do not depend on the angle of incidence, i.e. the absorption lines of the single dye layer are fixed at 485 nm and 520 nm wavelengths. The gap (or Rabi splitting) between LP and UP dispersion lines depends on the product of the dipole moments sum associated with organic dye exciton and the electric field of the plasmonic resonance per one vacuum oscillation. To accurately determine the interaction strength and its variation with the incident light angle, it is necessary to establish the dependence on optical dispersion. This involves determining the relationship of dispersion curves, the function of wave vector versus energy broadening (Figure 5).

**Figure 6:**
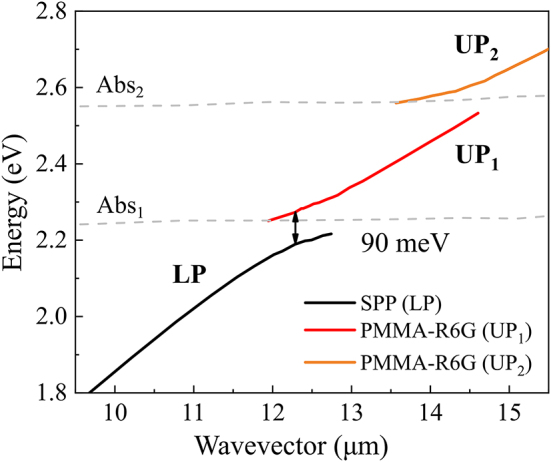
Dispersion relation showing the hybrid polariton modes (UP and LP) resulting from the strong coupling between surface plasmon polaritons (SPP) and the organic dye excitons (the absorption (Abs) lines of PMMA-R6G layer – grey dashed lines). The Rabi splitting of 90 meV is indicated. The observed states are hybrid excitations, not the parent SPP or exciton states.

When dye molecules strongly interact with plasmons, absorption lines shift to higher energy. This clearly shows the hybridization of dispersion lines and indicates a strong coupling regime and vacuum Rabi oscillations. The exact Rabi splitting for the hybrid plasmon-exciton polariton modes can be seen in Figure 6 and was determined to be about 90 meV.

The strong coupling regime in plasmonic-photonic structures is reached when the splitting of the modes (strong coupling parameter *g*) exceeds the energy losses (damping) [24] and the *g* parameter can be obtained only by the fitting of the whole spectra [23]. Yet the strong coupling regime can be easily distinguished from the weak coupling by observing the measured dispersion, where it can be defined by the splitting being large enough compared to the linewidths [28].

The wave vector value at the anti-crossing point (*k*_*x*_) was equal to 12.3 µm (taken from dispersion lines in Figure 2(b)). At this wave vector value, the corresponding energies of the upper (UP) and lower polariton (LP) branches were equal to 2.28 eV and 2.19 eV. From these energies and wave vector *k*_*x*_ the corresponding angles of incidence were calculated to reveal at what values the full width at half maximum (FWHM) for each UP and LP branch has to be evaluated in the experimental dispersion (Figure 2(b)). It was determined that the FWHM (damping *γ*) was equal to 120 meV (at 46.9° AOI and *E*_LP_ = 2.19 eV) and 90 meV (at 44.8° AOI and *E*_UP_ = 2.28 eV) for the LP and UP branches, respectively. Yet, to distinguish whether the strong coupling regime is reached, the coupling strength should exceed the linewidths of the coupled system, that is described by equation [24]:

$$g>\frac{1}{4}\left(\gamma_{pl}+\gamma_{em}\right),$$

where the right-hand side of this equation (damping) is equal to ∼52 meV. While in the weak coupling regime the mode damping prevails the light–matter interaction, the strongly coupled mode damping should be less than the Rabi gap [29]. This therefore shows the Rabi gap obtained in this research (90 meV) is larger than the damping (∼52 meV) of the hybrid mode, confirming that the system is in the strong coupling regime.

## 4 Summary

Summarising, this study employed total internal reflection ellipsometry (TIRE) to investigate the room temperature strong coupling between surface plasmon polaritons (SPP) and excitons within plasmonic-photonic nanostructures. Notably, the “filtered TIRE method” was applied for the first time in plasmon-exciton polariton system to experimentally verify strong coupling by selectively filtering exciton component in the hybrid polaritonic mode. The Rabi splitting, determined from the optical dispersion *E*(*k*) at the zero-detuning point, was measured to be 90 meV, the comparison of Rabi gap with the linewidths (*γ*) of the plasmonic and excitonic resonances, confirms the presence of strong coupling regime.

Fluorescence lifetime imaging microscopy (FLIM) has demonstrated that using polaritonic nanostructures where plasmons and excitons are in a strong coupling regime, reduces the fluorescence intensity by approximately 25 %, over the course of the 3 min measurement and the strongly coupled sample is photobleached approximately 6 times less. While fluorescence lifetime is in general independent of intensity, recovering accurate lifetimes (which in real systems are often bi-exponential in nature) requires a large number of photons [30]. Therefore, suppressing photobleaching could enhance FLIM measurements. The photobleaching can be suppressed by using plasmonic-photonic nanostructures supporting strong coupling and combining it with oxygen scavenging media. It was observed that the strong coupling between SPP and dye excitons plays a pivotal role in suppressing photobleaching, a phenomenon that often impedes fluorescence-based studies. The hybrid plasmonic-nanophotonic structures exhibit a remarkable ability to stabilize photo-bleaching mechanisms, thereby offering a promising avenue for the development of quantum multiparticle nanophotonic devices with organic molecules. Findings presented in this study offer possibilities for fundamental understanding of strongly coupled quantum dynamical systems, thus hold significant implications for various fields, including coherent light nanosources research, macroscopic quantum coherence, quantum biosensing and information processing.
